# Cerebroside C Increases Tolerance to Chilling Injury and Alters Lipid Composition in Wheat Roots

**DOI:** 10.1371/journal.pone.0073380

**Published:** 2013-09-13

**Authors:** Hong-Xia Li, Yu Xiao, Ling-Ling Cao, Xu Yan, Cong Li, Hai-Yan Shi, Jian-Wen Wang, Yong-Hao Ye

**Affiliations:** 1 College of Plant Protection, Jiangsu Key Laboratory of Pesticide Science, Nanjing Agricultural University, Nanjing, P. R. China; 2 Key Laboratory of Integrated Management of Crop Diseases and Pests, Ministry of Education, Nanjing, P. R. China; 3 College of Pharmaceutical Sciences, Soochow University, Suzhou, P. R. China; 4 School of Pharmacy, Jilin University, Changchun, P. R. China; Kansas State University, United States of America

## Abstract

Chilling tolerance was increased in seed germination and root growth of wheat seedlings grown in media containing 20 µg/mL cerebroside C (CC), isolated from the endophytic *Phyllosticta* sp. TG78. Seeds treated with 20 µg/mL CC at 4°C expressed the higher germination rate (77.78%), potential (23.46%), index (3.44) and the shorter germination time (6.19 d); root growth was also significantly improved by 13.76% in length, 13.44% in fresh weight and 6.88% in dry mass compared to controls. During the cultivation process at 4°C for three days and the followed 24 h at 25°C, lipid peroxidation, expressed by malondialdehyde (MDA) content and relative membrane permeability (RMP) was significantly reduced in CC-treated roots; activities of lipoxygenase (LOX), phospholipid C (PLC) and phospholipid D (PLD) were inhibited by 13.62–62.26%, 13.54–63.93% and 13.90–61.17%, respectively; unsaturation degree of fatty acids was enhanced through detecting the contents of CC-induced linoleic acid, linolenic acid, palmitic acid and stearic acid using GC-MS; capacities of superoxide dismutase (SOD), catalase (CAT) and glutathione peroxidase (GSH-Px) were individually increased by 7.69–46.06%, 3.37–37.96%, and −7.00–178.07%. These results suggest that increased chilling tolerance may be due, in part, to the reduction of lipid peroxidation and alternation of lipid composition of roots in the presence of CC.

## Introduction

Low temperature, an important environmental factor, constitutes one of the major yield and quality limitations to cereal productivity [1] and causes much injury in plants at the cellular level involving changes in malondialdehyde (MDA) content, reactive oxygen species (ROS) accumulation [2], membrane lipids composition and increases in activities of oxygen-scavenging enzymes [3] during the process of cold acclimation. On the other hand, there is a consensus that the plasma membrane was the primary site of injury and may be irreversibly dysfunctional due to a consequence of extra-cellular freezing [4]. The alternation to the lipid compositions seems to be responsible for the fate of the plasma membrane in chilling tolerance. In many plants, the increase in the proportion of unsaturated species of phosphatidylcholine and the degree of fatty acid unsaturation in the plasma membrane has a vital role in chilling tolerance [5], [6]. Changes in the structure and function of the plasma membrane were considered to be crucial for the manipulation of tissues metabolic processes inside the cell and even for the growth of plants under cold stress. Thus, there is an urgent need to discover cryoprotectant substances to protect cell membrane and to minimize cold-induced membrane rupture for altering crop growth and improving plants productivity under cold stress.

Endophytes are microorganisms that commonly reside within the plant tissues without causing any disease symptoms and have been recognized as a potential source of various novel active secondary metabolites with anticancer, antimicrobial, antiviral, antioxidant and other biological activities [7]–[9]. Besides, Meera *et al.* [10] reported that endophytic fungi including *Fusarium*, *Penicillium*, *Phoma* and *Trichoderma* could be designated as plant growth-promoting fungi and enhance the growth of a variety of crop plants. Many endophytes have been discovered to produce phenolic compounds to counteract ROS that escape the enzymatic antioxidant systems [11], and metabolize alkaloids to increase drought resistance to host plants [12]. More recently, some endophytic fungi have been recognized to improve growth of cucumber plants under salinity stress by producing many physiological active metabolites [8]. However, only little papers were reported on chilling resistance induced by endophytic fungi [13].

**Figure 1:**
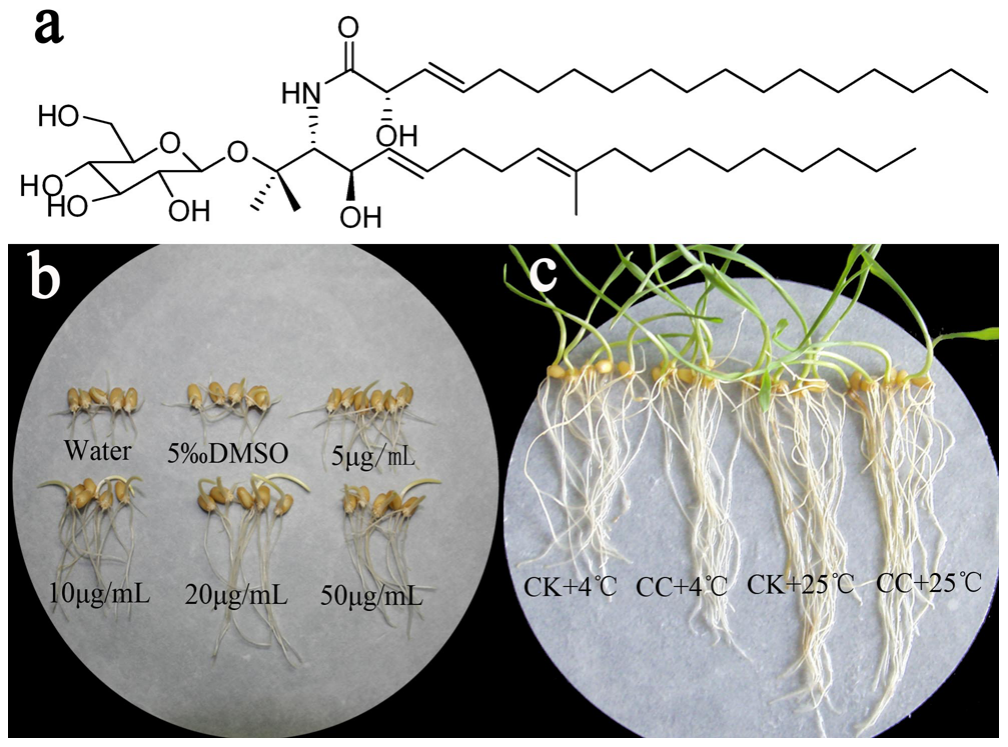
Effects of cerebroside C on seed germination and seedlings growth of wheat. **a**. Chemical structure of cerebroside C (CC) isolated from the strain TG78. **b**. The effects of cerebroside C at doses of 0, 5, 10, 20, 50 µg/mL on seed germination at 4°C stress for ten days. **c**. The promotion of cerebroside C at 20 µg/mL on root growth of wheat seedlings under cold stress. CC+4°C, treated with 20 µg/mL CC for three days at 25°C and followed three days at 4°C; CK+4°C, treated with 0.5% DMSO for three days at 25°C and followed three days at 4°C; CK+25°C, treated with 0.5% DMSO in growth chamber all the time.

In our previous research, an endophytic fungus *Phyllosticta* sp. (strain number TG78) was isolated from the stem of *Ginkgo biloba* which has long been used as a traditional medicine for various ailments in China [14]. Our preliminary experiments indicated that the fermented extract from *Phylosticta* sp. TG78 increases wheat seeds germination under cold stress. Based on the bioactivity-guided approach, two cerebrosides were ultimately isolated from TG78 for the first time and one of them (Figure 1a) was found to hold positive effects on wheat seedlings growth under chilling stress. Cerebrosides, isolated as natural elicitors [15], were a family of glycosphingolipids characterized as functional and dynamic components in flowing regulation of lipid bi-layer of cell membrane in fungi [16] and as inhibition to the phospholipase C-induced (PLC-induced) fusion of bilayer vesicles [17]. Some cerebrosides have been proven as elicitors to induce the synthesis of phytoalexins and pathogenesis-related proteins in rice and as mediators to regulate cell growth and stimulate cell morphogenesis [18]. In addition, cerebrosides assuming 9-methyl branched chain in yeast have an essential effect to maintain sufficient membrane fluidity for a low temperature environment [19]. Furthermore, in our previous studies, we found that cerebroside C (CC) had eliciting effects on biosynthesis of pharmaceutically important constituents such as taxol and artemisine in plant cells or hairy roots [20], [21]. However, all these studies have only addressed the effects of cererobrsides on the biotic stress and cell elicitation; information is currently lacking on physiological and biochemical changes provoked in wheat seedlings under low temperature with cererobrsides regarded as natural plant growth regulators. To assess how CC stimulated growth and improved chilling tolerance of early wheat seedlings, a detailed study of plant performance under chilling stress was carried out in this paper.

## Materials and Methods

### Fungal Material

The strain fungus TG78 (Genbank accession number KC445736) was isolated from the healthy sterilized leaf of *Ginkgo biloba* L. in Taixing, Jiangsu Province, P.R. China and was identified as *Phyllosticta* sp. based on the morphological properties and the DNA sequence of the ITS1-5.8S-ITS2 [22].

### Fermentation, Fractionation and Purification of Cerebrosides

The preparation of liquid inoculum and the subsequent solid fermentation of the fungus TG78 were accomplished according to Song *et al.* [23] The extract of fermentation was harvested after air-dried naturally, subsequently marinated with CHCl_3_-CH_3_OH gradient (1∶1 v/v) for 24 h at room temperature, and then filtered and evaporated under reduced pressure for three times.

In order to separate active compounds, the extract was further suspended in 500 mL of water and extracted successively with 500 mL petroleum ether, 500 mL ethyl acetate, 500 mL *n*-butanol for three times respectively at room temperature to give a petroleum ether fraction (A, 115.1 g), ethyl acetate fraction (B, 4.7 g) and a *n*-butanol fraction (C, 15.1 g). Based on the activity-guided approach, the petroleum ether phase precipitated the salts and lipids was chromatographed on silica gel (200 g, 100–200 mesh) using a stepwise gradient of CHCl_3_/CH_3_OH (100 : 0→0 : 100 v/v) to give five fractions (A_1_, 0.9 g; A_2_, 2.5 g; A_3_, 3.6 g; A_4_, 1.3 g; A_5_, 2.8 g) based on TLC at 254 nm, spraying with 10% H_2_SO_4_-EtOH followed by heating at 100°C for 10 min. Fraction A_2_ was then separated on a Sephadex LH-20 column eluted with CHCl_3_/CH_3_OH (1∶1 v/v), followed by recrystallization to yield a metabolite (80 mg), which was established as (2S,2′R,3R,3′E,4E,8E)-1-O-β-D-glucopyranosyl-2-N-(2′-hydroxy-3′- octadecenoyl) -3-hydroxy-9-methyl-4,8-sphingadienine (cerebroside C, CC) (Figure 1a) based on ^1^H-NMR, ^13^C-NMR, ^1^H-^1^H COSY, HMBC, HSQC and ESI-MS corresponded to that reported by Shu *et al.* [24] In a similar way, cerebroside B (20 mg) in *n*-butanol fraction was purified, but not selected for further study considering its limited quantity.

### Elicitation Treatments on Seed Germination

A stock solution of CC at 500 µg/mL in 5% dimethyl sulfoxide (DMSO) was serially diluted up to the desired final concentrations (5, 10, 20, and 50 µg/mL). The vehicle (0.5% DMSO in water) was used as control treatment. All the final DMSO concentration of the diluted solution was kept at 0.5% (v/v) to ensure that DMSO at the concentration did not interfere with the experiments.

Seeds of wheat (*Triticum aestivum*, cv. Huaimai 25), obtained from Huaiyin Institute of Agricultural Sciences, Huai'an, Jiangsu Province, P. R. China, were surface sterilized for 10 min in 5% NaClO solution, rinsed thoroughly and germinated on filter papers with distilled water in Petri dishes for 24 h at 25°C in dark. Thereafter, three replicates of 28 equal-sized and plump germinating seeds were selected to place on new filter paper moistened with CC aqueous solution and sprayed only 0.5% DMSO for control seedlings (CK) for 10 days at 4°C under cold stress treatment. All the seeds were watered daily with appropriate aqueous solution of CC or 0.5% DMSO to keep the filter papers moistened. Data on germinant seeds of different treatments was recorded at 24 h intervals as radical emergence to 3–5 mm and the effects on wheat growth under low temperature were determined by calculating germination rate (GR), germination potential (GP), germination index (GI) and the mean germination time (MGT) [25].
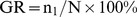


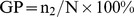


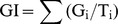


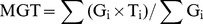
n_1_: the total number of germinated seeds at the tenth day; n_2_: the total number of germinated seeds at the third day; N: the whole number of tested seeds; G_i_: the number of germinated seeds on day T_i_; T_i_: the corresponding germinant days.

### Elicitation Treatments on Seedling Growth

The germination, sowing and CC treatments were consistent with those stated above except the culture conditions. The culturing procedures were as follows: (1) Before cold stress, all germinated wheat seeds were cultured in the growth chamber at approximately 25°C for 3 days ensuring sufficient available sample of roots. (2) Cold stress, that is, on the 4th day, the wheat seedlings of control were evenly divided into two groups. One group of CK (CK+4°C) and CC treatments (CC+4°C) were transferred to a temperature of 4°C for another 3 days, while the other seedlings of CK and CC were still kept in the growth chamber as room temperature treatment (CC+25°C) and room temperature control (CK+25°C) for the same time. (3) Recovering room temperature, after chilling stress treatment, the plants at 4°C were recovered to the same conditions of the last three days with those at 25°C. A 12/12 h (day/night) photo-period and a light intensity of 6000 lx were maintained during the nine days. Then some groups grown of the three periods (before cold stress, cold stress and recovering room temperature) were measured for the morphological characters and growth parameters of roots and shoots, while the other samples at the time of 0, 6, 12, 24, 48, and 72 h in cold stress for three days, together with those recovering to room temperature for 24 h (96 h in total) were harvested for physiological targets analysis. Three replicates (n = 3) were done for each condition, and commonly 5–10 seedlings were used to provide enough amounts of plant material to measure growth parameters and physiological targets analysis in each replicate.

### Analysis of Lipid Peroxidation and Relative Membrane Permeability

Lipid peroxidation was determined as the amount of malondialdehyde (MDA) based on the spectrophotometer measurement of a red-complex produced during the reaction of thiobarbituric acid (TBA) with MDA as described earlier [26]. In brief, fresh roots (0.1 g, fresh weight, FW) were ground under liquid nitrogen, and suspended in 0.9 mL extracted solution containing 50 mM phosphate buffer (pH 7.4) and 1% polyvinylpyrrolidone (PVP). Then the homogenate was centrifuged at 4°C, 3500 rpm for 10 min to collect the supernatant, which was further processed following the instruments of MDA assay kit (for plant) purchased from Nanjing Jiancheng Bioengineering Institute (Nanjing, China). Finally, the absorbance of red TBA-MDA complex was measured using a microplate reader (SpectraMax M5, USA) at 532 nm and the MDA content (nmol/g FW) was calculated according to detailed instructions of the MDA assay kit.

The relative membrane permeability (RMP) was determined based on Yang *et al.* [27] The roots were excised (5 mm length), placed in 15 mL of deionised distilled water and then vortex for 5 s for initial electrical conductivity (EC_0_) using a conductivity meter (CON-510, Singapore). After immersed for 10 h at 15°C, the solution was then assayed for EC_1_. The same samples containing roots were autoclaved at 100°C for 30 min to determine EC_2_. Percent RMP was calculated as:
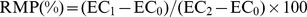
5 to 10 seedlings were used to provide enough amounts of root tissues for MDA and RMP in each experimental replicate (n = 3).

### Analysis of membranous phospholipid-catabolizing enzymes

The enzyme extract of lipoxygenase (LOX, EC 1.13.11.12), phospholpase C (PLC, EC 3.1.4.3) and phospholpase D (PLD, EC 3.1.4.4) were extracted according to the method described by Rui *et al.* [28] In brief, fresh roots (0.2 g) were ground under liquid N_2_, and then 5 mL of 50 mM tris-HCl (pH 8) was added, containing 10 mM KCl, 500 mM sucrose and 0.5 mM phenylmethylsulfonylfluoride. The extracts were then homogenised and centrifuged at 12000× g for 10 min at 4°C. One unit of LOX was defined as the amount of enzyme causing an increase in absorption of 0.01 per min at 234 nm and 25°C when linoleic acid is employed as the substrate. PLC and PLD assay was determined according to Munishwar and Finn [29]. One unit of PLC was defined as the amount of enzyme that catalysed the formation of 1 nmol D-nitrophenol/h, the same as PLD. 5 to 10 seedlings were used to provide enough amounts of root tissues for LOX, PLC and PLD in each experimental replicate (n = 3).

### Extraction and Determination of Fatty Acids

Fatty acids were extracted, methanol etherified and analyzed according to Hassan *et al.* [30]. The fresh roots (0.3 g) of wheat seedlings collected respectively at appropriate time were homogenized in 6 mL of chloroform : methanol : water (1 : 2 : 0.8) and extracted in ultrasonic water bath for 45 min before filtering and then stand for 30 min after adding 1 mL of 0.78% NaCl. The organic phase was collected and taken to dryness by N_2_. Methylation of fatty acids was carried out by adding 5 mL of 0.5 M KOH methanol for 40 min at 50°C under N_2_. The resultant fatty acid methyl esters (FAME) were extracted twice with high performance liquid chromatography-grade hexane and determined by GC-MS.

GC-MS analyses were performed by an Aligent series 6890 capillary gas chromatography - mass spectrometry (GC-MS) equipped with a J&W DB-1701 MS fused-silica capillary column (30.0 m length×0.25 mm i.d.×0. 25 µm film thickness) under programmed conditions: 140°C for 1 min then rising at 4°C per min to 164°C and then at 15°C per min to 180°C, and finally at 5°C per min to 235°C for 1 min. Helium was used as a carrier gas which flowed at a rate of 1 mL/min. The injection volume was 1 µl, and the temperature of the detector and injector was 280°C and 250°C, respectively. The FAME determination was identified by MS (Figure S1) and valued through the external standard curve method. 5 to 10 seedlings were used to provide enough amounts of root tissues in each experimental replicate (n = 3).

### Extraction and Measurement of Antioxidant Enzymes

For enzyme extracts and assays, fresh roots (0.1 g) were ground in liquid nitrogen, and then suspended in 0.9 mL solution containing 10 mM phosphate buffer (pH 7.4). The homogenate was centrifuged at 4°C, 2500 rpm for 10 min and the resulting supernatant was collected for determination of the activities of superoxide dismutase (SOD, EC 1.15.1.1), catalase (CAT, EC 1.11.1.6), peroxidase (POD, EC 1.11.1.7) and glutathione peroxidase (GSH-Px, EC 1.11.1.9) using commercial assay kits purchased from Nanjing Jiancheng Bioengineering Institute (Nanjing, China). All enzymes above were detected using a microplate reader (SpectraMax M5, USA), and 5 to 10 seedlings were used to provide enough amounts of root tissues in each experimental replicate (n = 3).

The activity of SOD was determined by measuring the inhibiting rate of the enzyme to O_2_^−^· produced by the xanthine morpholine with xanthine oxidase using the SOD assay kit. Each endpoint assay was detected the red substances of the reaction system by absorbance at 550 nm after 40 min of reaction time at 37°C. And one unit SOD activity (U) was defined as the quantity of SOD required to produce 50% inhibition of reduction of nitrite in 1 mL reaction solution by measuring the change of absorbance at 550 nm.

The CAT activity was measured based on the hydrolysis reaction of hydrogen peroxide (H_2_O_2_) with CAT, which could be terminated by molybdenum acid (MA) to produce yellow MA-H_2_O_2_ complex. CAT activity was calculated by the decrease in absorbance at 405 nm due to the degradation of H_2_O_2_, and one unit is defined as the amount of enzyme that will cause the decompose of 1 µmol hydrogen peroxide (H_2_O_2_) per second at 37°C in 1.0 g fresh tissue according to CAT assay kit.

The POD activity was measured based on the change of absorbance at 420 nm by catalyzing H_2_O_2_. One unit was defined as the amount of enzyme which was catalyzed and generated 1 µg substrate by 1.0 g fresh tissues in the reaction system at 37°C. POD activity was calculated as the formula according to POD assay kit.

The GSH-Px activity was also measured using the assay kit based on the principle that oxidation of glutathione (GSH) and hydrogen peroxide (H_2_O_2_) could be catalyzed by GSH-Px to produce oxidized glutathione (GSSG) and H_2_O. In addition GSH reacts with 5, 5′-dithiobis (2-nitrobenzoic acid) (DTNB) to produce stable yellow substances and the decrease of GSH at 412 nm during the reaction is indicative of GSH-Px activity in tissues. One GSH-Px unit of GSH-Px activity (U) was calculated as the amounts of enzyme that will oxidize 1 µmol/L GSH in reaction system at 37°C per minute in 1.0 g fresh tissue according to the assay kit. All of the enzymes were expressed as in U/g FW.

### Statistical Analysis

All the experiments were conducted in triplicate (n = 3) and statistical analysis of the data from the control and the chill-stressed plants was performed by analysis of variance (One-Way ANOVA), using SPSS 16.0 software. A probability value of p≤0.05 was considered to denote a statistically significance difference. Data are presented as mean ± standard deviation (SD) of three replicates.

## Results

### CC Induces Seed Germination

At low temperatures, germination percentage of wheat seed was lower. To investigate whether CC could promote germination under chilling stress, a preliminary experiment at 4°C with CC at 0, 5, 10, 20, and 50 µg/mL was carried out. It was also to determine the optimal dosage showing a maximal effect. As demonstrated in Table 1 and Figure 1b, 10 days after planting, the response of the seeds to various doses of CC showed higher GR of 64.20–77.78%, better GP of 8.64–23.46% and higher GI of 2.79–3.44, but much shorter MGT of 6.19–6.94 d compared with that of controls (60.50%, 6.17%, 2.73, and 7.13 d, respectively). GR, GP, GI and MGT peaked at 20 µg/mL of CC (77.78%, 23.46%, 3.44, and 6.19 d, respectively) with significant difference from controls. Whereas a slightly decrease was measured at the higher concentration (50 µg/mL). Thus, the optimal dose (20 µg/mL) was selected as the CC concentration for the subsequent study.

**Table 1:** Effects of cerebroside C (CC) at 4°C on seed germination of wheat.

CC content (μg/mL)	Germination rate (%)	Germination potential (%)	Germination index	Mean germination time (d)
0	60.50±4.30^a^	6.17±2.1^a^	2.73±0.31^a^	7.13±0.10^a^
5	64.20±4.28^a^	8.64±2.1^b^	2.79±0.33^a^	6.94±0.27^ab^
10	76.54±2.14^b^	12.35±2.1^c^	3.14±0.08^b^	6.78±0.20^b^
20	77.78±3.70^b^	23.46±4.3^d^	3.44±0.10^c^	6.19±0.12^c^
50	75.31±2.41^b^	7.41±2.6^a^	3.25±0.09^b^	6.90±0.14^ab^

0 = 0.5% DMSO distilled water. In each column, the different letter indicates significant (p≤0.05) difference as evaluated by Duncan's Multiple Range Test (DMRT). Results are expressed as the mean (±) standard deviation (SD) of three replicates (n = 3, 10 seedlings in each replicate).

### CC Stimulates Seedlings Growth

To investigate the elicitation of CC on the wheat seedlings growth, an experiment with three culturing procedures, before cold stress, cold stress and recovering room temperature, were carried out. As shown in Figure 1c and Table 2, CC treatment at room temperature (CC+25°C) had little effect on root or shoot growth of wheat seedlings while CC improved the growth parameters and biomass accumulation in terms of roots length, roots fresh weight and dry weight (P≤0.05) after cold stress (CC+4°C) and recovering to room temperature. The root length, fresh and dry weight of CC-inoculated seedlings were 8.60 cm, 45.08 mg and 5.75 mg after cold stress, increased by 13.76%, 13.44% and 6.88% compared to those of non-CC plants under 4°C. However, CC had few functions on the growth of shoots during these procedures. Both the root of seedlings and 4°C stress were thus chosen as the object and the major condition for the subsequent study.

**Table 2:** Effects of cerebroside C at 20 µg/mL on seedlings growth of wheat.

Treatments	Before low temperature stress		After low temperature stress		Recovering to room temperature	
	Root length (cm)	Shoot height (cm)	Root length (cm)	Shoot height (cm)	Root length (cm)	Shoot height (cm)
CK+4°C	7.49±0.23^a^	5.32±0.24^a^	7.56±0.15^a^	5.68±0.22^a^	7.76±0.13^a^	7.57±0.40^a^
CC+4°C	7.62±0.16^a^	5.31±0.25^a^	8.60±0.15^b^	6.08±0.22^b^	8.92±0.25^b^	7.56±0.32^a^
CK+25°C	7.49±0.23^a^	5.32±0.24^a^	10.26±0.09^c^	8.46±0.12^c^	10.88±0.33^c^	8.94±0.38^b^
CC+25°C	7.62±0.16^a^	5.31±0.25^a^	10.36±0.15^c^	8.43±0.12^c^	11.18±0.01^c^	9.08±0.38^b^

Treatments	Before low temperature stress		After low temperature stress		Recovering to room temperature	
	Root fresh weight (mg/root)	Shoot fresh weight (mg/shoot)	Root fresh weight (mg/root)	Shoot fresh weight (mg/shoot)	Root fresh weight (mg/root)	Shoot fresh weight (mg/shoot)
CK+4°C	39.60±1.23^a^	41.83±3.48^a^	39.74±1.00^a^	44.23±2.02^a^	42.88±1.03^a^	51.98±2.93^a^
CC+4°C	41.28±2.07^a^	41.55±4.46^a^	45.08±3.89^a^	46.67±1.99^a^	52.70±4.43^b^	52.50±4.73^a^
CK+25°C	39.60±1.23^a^	41.83±3.48^a^	52.98±3.86^b^	58.91±0.63^c^	57.92±1.73^c^	71.04±5.19^b^
CC+25°C	41.28±2.07^a^	41.55±4.46^a^	50.29±1.13^b^	55.52±0.78^b^	58.84±1.20^c^	70.74±3.81^b^

Treatments	Before low temperature stress		After low temperature stress		Recovering to room temperature	
	Root dry weight (mg/root)	Shoot dry weight (mg/shoot)	Root dry weight (mg/root)	Shoot dry weight (mg/shoot)	Root dry weight (mg/root)	Shoot dry weight (mg/shoot)
CK+4°C	5.32±0.03^a^	5.21±0.22^a^	5.38±0.36^a^	6.61±0.50^a^	5.48±0.15^a^	7.51±0.33^a^
CC+4°C	5.39±0.04^a^	5.05±0.12^a^	5.75±0.23^b^	6.63±0.16^a^	6.07±0.09^b^	7.50±0.21^a^
CK+25°C	5.36±0.07^a^	5.21±0.22^a^	6.59±0.29^c^	8.97±0.13^b^	6.96±0.23^c^	9.40±0.64^b^
CC+25°C	5.39±0.04^a^	5.05±0.12^a^	6.75±0.17^c^	8.89±0.18^b^	7.02±0.04^c^	9.37±0.29^b^

In each column, the different letter indicates significant (p≤0.05) difference as evaluated by Duncan's Multiple Range Test (DMRT). Results are expressed as the mean (±) standard deviation (SD) of three replicates (n = 3, 10 seedlings in each replicate).

### Effects of CC on Membrane Injury in Wheat Seedlings

**Figure 2:**
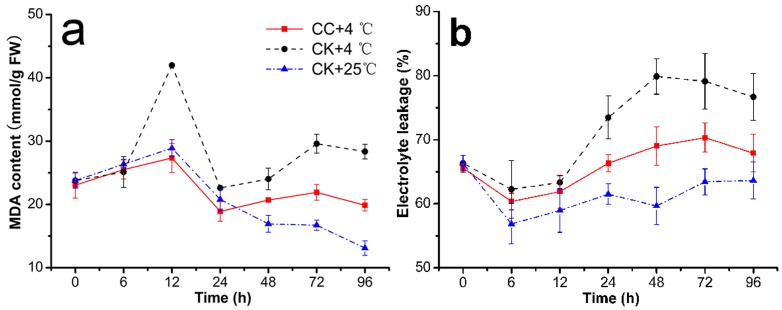
Impact of cerebroside C on MDA and RMP. This was observed in roots of wheat seedlings during 4°C stress at 0, 6, 12, 24, 48, 72 h and recovering 25°C for 24 h (total 96 h). The results were expressed as the mean of three replicates (n = 3, 0.1 g fresh roots from 5–10 seedlings in each replicate). **a**. MDA, malondialdehyde content in 0.1 g roots of wheat seedlings was demonstrated by assay kit purchased from Nanjing Jiancheng Bioengineering Institute (Nanjing, China); **b**. RMP, relative membrane permeability was estimated using biochemical assays. CC+4°C, treated with 20 µg/mL CC under cold stress; CK+4°C, treated with 0.5% DMSO under cold stress; CK+25°C, treated with 0.5% DMSO in growth chamber at 25°C.

Membrane injury, expressed as the MDA content and the relative membrane permeability (RMP) value, worsened as low temperature of 4°C went on. CC treatments at chilling stress considerably affected the MDA content and relative membrane permeability (RMP) in roots of wheat seedlings, which are given in Figures 2a and 2b. MDA content of CC-treated seedlings was decreased by −1.63–29.91% compared to cold control (CK+4°C), expressing significant differences from 12 h to 96 h and even approaching to the level of room temperature control at 12 h (41.99, 27.33, 28.90 mmol/g FW; CK+4°C, CC+4°C, CK+25°C) (Table S1). Cold stress also increased membrane leakage in roots of CC-treatment (CC+4°C) and cold control (CK+4°C) from 12 h to 72 h compared to those in control groups at 25°C (CK+25°C), but (CC+4°C) expressed the lower values than that of (CK+4°C), and there were no significant differences among the three treatments (Table S2) from 24 h to 72 h.

**Figure 3:**
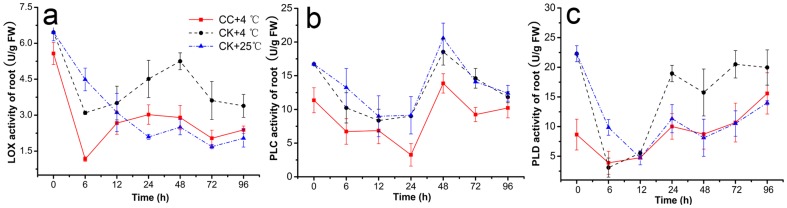
Inhibition of cerebroside C on the activities of LOX, PLC and PLD. This was observed in roots of wheat seedlings during 4°C stress at 0, 6, 12, 24, 48, 72 h and recovering 25°C for 24 h (total 96 h). The results were expressed as the mean of three replicates (n = 3, 0.2 g fresh roots from 5–10 seedlings in each replicate). **a**. Activities of lipoxygenase (LOX), **b**. phospholipid C (PLC) and **c**. phospholipid D (PLD) in roots of wheat seedlings were determined individually using biochemical assays. CC+4°C, treated with 20 µg/mL CC under cold stress; CK+4°C, treated with 0.5% DMSO under cold stress; CK+25°C treated with 0.5% DMSO in growth chamber at 25°C.

In addition, this study attempted to associate the alleviation of chilling injury by CC with the inhibited activities of LOX, PLC and PLD. As we all known, PLD, PLC together with LOX, related to lipid unsaturation level and cell membrane integrity, plays an important role in generating phospholipd degradation and peroxidative damage in membrane lipids under cold stress [31], [32]. As shown in Figures 3a and 3c, the activities of LOX and PLD varied with similar tendency in (CC+4°C) and (CK+4°C) treatments, which decreased at earlier 6 h and following an increasing order after that. With the significantly lower activities by 29.50–44.76% (Table S3) and 21.88–47.90% (Table S5) of (CC+4°C) than that of (CK+4°C) from 24 h to 96 h, LOX and PLD in (CC+4°C) even approached to the level of (CK+25°C). However, PLC activity (Figure 3b) expressed a variation trend of decreasing before 24 h and then increasing at 48 h, with significantly lower level by 13.54–63.93% in (CC+4°C) than (CK+4°C) (Table S4).

### Effects of CC on Fatty Acid Content in Roots

**Figure 4:**
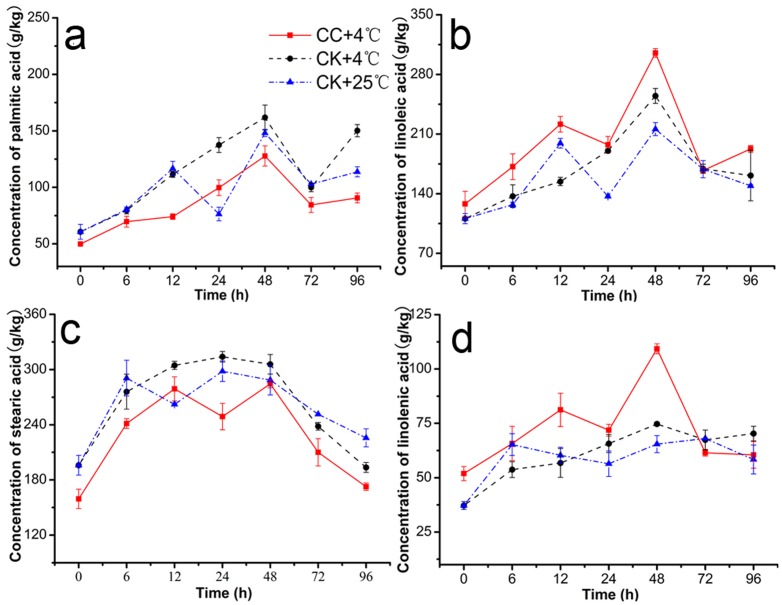
Effects of cerebroside C on contents of C16:0, C18:2, C18:0, C18:3. This was observed in roots of wheat seedlings during 4°C stress at 0, 6, 12, 24, 48, 72 h and recovering 25°C for 24 h (total 96 h). The results were expressed as the mean of three replicates (n = 3, 0.3 g fresh roots from 5–10 seedlings in each replicate). **a**. Palmitic acid (C16:0), **b**. linoleic acid C18:2 (2), **c**. stearic acid C18:0 (3) and **d**. linolenic acid C18:3 in roots of wheat seedlings was identified by MS and valued through the external standard curve method. CC+4°C, treated with 20 µg/mL CC under cold stress; CK+4°C, treated with 0.5% DMSO under cold stress; CK+25°C treated with 0.5% DMSO in growth chamber at 25°C.

Significant changes were observed both in saturated and unsaturated fatty acids during 4°C stress and recovering 25°C for 24 h (Figure 4). The linoleic acid (C18:2) (Figure 4b) and linolenic acid (18:3) (Figure 4d) contents in the roots of (CC+4°C) and (CK+4°C) increased compared to those of controls (CK+25°C) and the content of the palmtic acid (C16:0) (Figure 4a) and stearic acid (C18:0) (Figure 4c) decreased in CC-treated group, however, there were almost no significant differences in the content of C18:0 among the there treatments. At 12 h and 48 h under cold stress, C18:2 contents in (CC+4°C) considerably increased by 43.65% and 19.82% over control (CK+4°C) (Figure 4b) and that of C18:3 increased by 43.03% and 46.29% (Figure 4c). On the contrast, the C16:0 contents decreased by 50.51% and 26.66% over (CK+4°C) (Figure 4a). Thus, the ratio of unsaturated to saturated fatty acids [(18:2+18:3)/(16:0+18:0)] at these two time also showed that a significant increase in CC-treated roots, which can be regarded as qualitative evidence of CC involving alterations in fatty acid unsaturation degree. It is important to note that the change in the unsaturation of the membrane phospholipids is associated with their functional activity.

### Effects of CC on Activities of Antioxidant Enzyme in Roots

**Figure 5:**
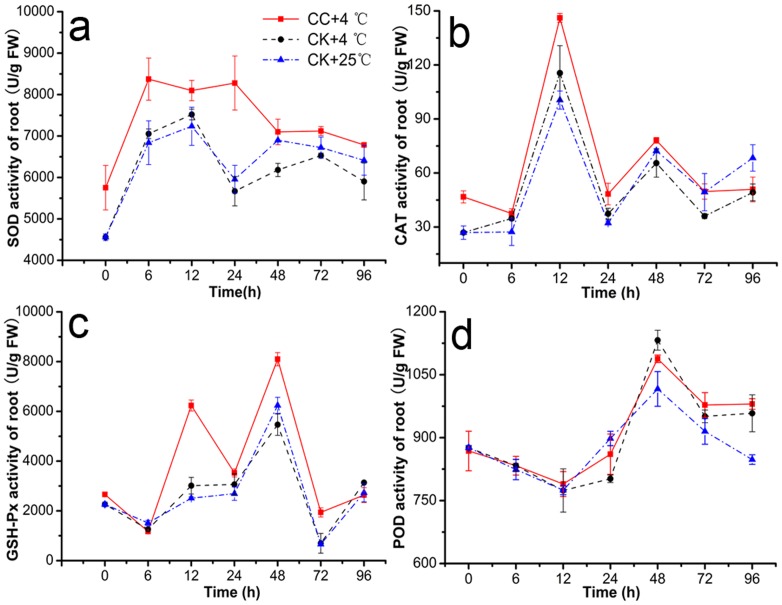
Antioxidant enzyme activities. This was observed in roots of wheat seedlings during 4°C stress at 0, 6, 12, 24, 48, 72 h and recovering 25°C for 24 h (total 96 h). Results were expressed as the mean of three replicates (n = 3, 0.1 g fresh roots from 5–10 seedlings in each replicate). Activities of **a**. superoxide dismutase (SOD), **b**. catalase (CAT), **c**. glutathione peroxidase (GSH-Px) and **d**. peroxidase (POD) in roots of wheat seedlings were examined using assay kits purchased from Nanjing Jiancheng Bioengineering Institute (Nanjing, China). CC+4°C, treated with 20 µg/mL CC under cold stress; CK+4°C, treated with 0.5% DMSO under cold stress; CK+25°C treated with 0.5% DMSO in growth chamber at 25°C.

Antioxidant enzyme activities of SOD, CAT, POD, and GSH-Px in CC treatments were increased significantly in roots under cold stress and generally presented to be firstly increased and then decreased in roots of stressed plants (Figure 5). Plus, the contents of (CK+4°C) were less than that of (CC+4°C) except POD, and notably different at least at one check point (6, 12, 24, or 48 h). Under low temperature, CC raised SOD activity by 7.69–46.06% compared to (CK+4°C) (Figure 5a). At 6 h and 24 h, the effects were significantly enhanced by 1.19 and 1.46 -folds, respectively. In addition, ANOVA indicated that CAT activity in roots was improved by 3.37–37.96% in (CC+4°C) treatments (P≤0.05) under 4°C, and significantly peaked 37.96% at 72 h. But little obvious differences at other time among the three treatments were observed (Figure 5b). Similarly, GSH-Px activity was significantly increased by −7.00–178.07% in (CC+4°C) treatments and at 12 h and 48 h, the activity was enhanced by almost 2-fold (Figure 5c). However, CC treatments did not change the POD activity notably as shown in Figure 5d. One-Way ANOVA of SOD, CAT, POD, and GSH-Px could be found in Tables S10, S11, S12, S13.

## Discussion

Previous studies have demonstrated that cerebrosides isolated from a range of fungal pathogens including *Fusarium oxysporum*, *Pythium* sp. and *Botrytis* sp. possessed potent elicitor activity [21]. Cerebrosides assuming 9-methyl branched chain had an essential effect to maintain sufficient membrane fluidity for a low temperature environment [18]. In addition, asteriacerebrosides A, regarded as a plant-growth promoter, exhibited a root stimulating effect in *Brassica campestris* L. [33]. Furthermore, CC could induce the activity of phenylalanine ammonla-lyase (PAL), improve nitric oxide burst to regulate the biosynthesis of taxol [20] and stimulate artemisine synthesis in combination with nitric oxide elicitation [21]. Some of these responses occurred in our study. More importantly, our study showed that CC possessed the growth-promoting activity under cold stress. The treatments with CC exhibited higher seed germination percentage, more upstanding growth and other responses in wheat seedlings compared to the control. CC as a novel natural plant-growth regulator under chilling stress is therefore interesting and most certainly noteworthy.

Chilling stress has effects ranging from morphological to molecular levels and the effects are evident at all physiological stages of plant growth. Wheat is sensitive to prolonged exposure to low temperature particularly at earlier seedlings growth, which can result in significant chilling injury and mortality [34]. In our experiments, compared to the control, CC-treatments had increases in germination rate, potential and index of 28.56%, 280.23% and 26.00%, respectively, and also decreased the mean germination time of 15.19% in CC-treatment undergoing chilling stress. Furthermore, CC improved significantly root elongation and then root biomass compared with controls, but few effects on the shoots. In addition, at temperature of 25°C, CC had insignificant effects both on roots and shoots. In other words, CC showed the activity of promoting germination process, keeping emergence consistent and accelerating the growth of roots in wheat to some extent under low temperature conditions (Tables 1 and 2).

Chilling stress usually causes injury such as the ROS formation in plant cells, which seriously damage membrane lipids, proteins and nucleic acids and disturb the homeostasis of the organism [35], [36]. And low-temperature effect on cellular membrane is the direct result that causes a slowdown of metabolism, solidification of cell membranes and loss of membrane functions. The excessive accumulation of ROS can induce destructive oxidative processes leading to a loss of permeability control and an increase of MDA content in tissue, which could reflect the degree of lipid peroxidation and could be good indicators of the structural integrity of the membranes of plants subjected to chilling stress [37]. In our study, low temperature treatments with CC expressed lower relative membrane permeability and MDA content in roots than the controls (Figure 2a), thus exhibiting decreased degree of membrane breakage (Figure 2b). That is to say that CC had a stronger lipid peroxidation inhibitory effect especially at 12 h of 4°C cold stress and kept the effect until 96 h (recovering 25°C for 24 h).

Interestingly, CC induced long-term protection against cold stress in wheat seedlings by affecting activities of the main antioxidant enzymes such as SOD, CAT, POD, and GSH-Px (Figure 5), which are responsible jointly for alleviating or preventing low-temperature-induced oxidative injury such as the ROS formation in plants [38]. SOD forming the first defendable line against ROS under stress [39] could clean the harmful substance to cell membrane superoxide (O^2−.^), and the SOD activity was induced firstly by CC in our study at the early time (6–24 h) under chilling stress and expressed the similar variable trend with the results of Ferrareze *et al.* [40] Following, the end product of SOD, hydrogen peroxide (H_2_O_2_) could be metabolized into oxygen and water by CAT [41], which also expressed much higher activity at 12 h in CC-treatments. Whereas, GSH-Px, another important enzyme for removing H_2_O_2_, could also repair other peroxides such as fatty acids and phospholipids peroxides [42], [43], and significant higher activity was found at 12 h and 24 h during cold stress CC-treatment. With the same changeable pattern of these antioxidant enzymes as previously shown in sweet potato and tomato under chilling stress [44], [45]. Our results showed that CC might alleviate the antioxidant damage to cell membrane in roots through cleaning over more ROS by higher activities of enzymes such as SOD, CAT, and GSH-Px at the early time of cold stress. However, it is noticeable that POD was an exception; it may because that POD has a similar mechanism of CAT to scavenge ROS while the majority of reactive oxygen was removed by CAT, leading to the maintenance of POD activity compared to controls [46].

On the other hand, more lipolytic cascades achieved by the concerted membranous phospholipid-catabolizing enzymes including LOX, PLC, and PLD [47], [48] could directly result in a lack of membrane stability, and have been long known to be major contributors to chilling-induced membrane damage in plants [49]. And the composition change in membrane lipids could also be expressed by the decrease in unsaturation of lipid fatty acids and bulk membrane lipid phase transitions [50]. The enzymes of LOX, PLC and PLD are thought to play important roles in generating phospholipid degradation and peroxidative damage in membrane lipids, and higher activities of these enzymes will result in decreased level of lipid unsaturation, lipid fruity and cell membrane integrity during cold stress. In our study, all of these enzymes in CC-treatments (CC+4°C in Figure 3) maintained lower activities than those of CK-treatments (CK+4°C in Figure 3). Especially the activities of LOX and PLD were obviously inhibited by CC from 24 h at 4°C. In addition, the fatty acid unsaturated degree of CC-treatments was also effectively enhanced in response to the chilling stress through increasing the linoleic acid (C18:2) and linolenic acid (18:3) contents and decreasing palmitic acid (C16:0) and stearic acid (C18:0) contents compared to those of controls (Figure S1; Tables S6, S7, S8, S9), which may alleviate injury of membrane bilayer phase and higher degree of unsaturated fatty acids in response to cold stress in roots of CC-treatments. These results were similar to previous studies which reported that the development of chilling injury in cucumber and grape berry was accompanied with increases of LOX and PLD activities under chilling stress [51], [52], and that membrane lipids from chilling-resistant plant species showed higher content of unsaturated fatty acids than did sensitive species [53], [54]. Taken LOX, PLC, PLD activities and the degree of unsaturated fatty acids together, present work seems to suggest that the alleviation of chilling injury in CC-treatments is clearly related with inhibition of these enzymes.

In this sense, the decrease in the level of MDA at earlier time of cold stress (before 12 h) might result from improved activities of SOD, CAT, and GSH-Px by CC, leading to the alleviation in relative membrane permeability later (from 24–96 h). At the same time, the increase in contents of C18:2, C18:3 and the degree of unsaturated fatty acids might attributed in inhibition of activities of LOX, PLC and PLD induced by CC, and finally increasing the tolerance of wheat seedling under cold stress.

In conclusion, the promotion of CC on the seed germination and growth of wheat seedlings under cold stress might due to maintain the integrity and stability of membrane under chilling stress by inhibiting the hydrolyzing enzymes of LOX, PLC and PLD, enhancing the unstauration degree of fatty acids, and increasing the antioxidant enzymes activities of SOD, CAT, and GSH-Px. Our present study suggested that CC may be employed as a kind of natural plant growth regulators. Further investigations about the mechanism of CC on the wheat seedlings and whether cerebroside C together with other cerebrosides, including cerebroside B, in extract from *Phyllosticta* sp. TG78 synergically promoting the germination of wheat seeds under cool conditions are required to be fully elucidated.

## Supporting Information

Figure S1 **Detection of fatty acids (C16:0, C18:2, C18:0, C18:3) in roots of wheat seedlings at 48 h under cold stress by GC-MS.** **a**. GC-MS total ion chromatogram (TIC); **b**. mass spectrum of the methyl esters of palmitic acid C16:0 (1), linoleic acid C18:2 (2), stearic acid C18:0 (3) and linolenic acid C18:3 (4) in roots of wheat seedlings at 48 h of cold stress. CC+4°C, pretreated with 20 µg/mL CC under cold stress; CK+4°C, treated with 0.5% DMSO under cold stress; CK+25°C, treated with 0.5% DMSO in growth chamber at 25°C. Results are expressed as the mean of three replicates (n = 3) derived from 5–10 seedlings.
(TIF)

Table S1 Effects of cerebroside C (20 µg/mL) on MDA content in roots of wheat seedlings under cold stress (4°C).
(DOC)

Table S2 Effects of cerebroside C (20 µg/mL) on RMP value in roots of wheat seedlings under cold stress (4°C).
(DOC)

Table S3 Inhibition of cerebroside C (20 µg/mL) on activity of LOX in roots of wheat seedlings under cold stress (4°C).
(DOC)

Table S4 Inhibition of cerebroside C (20 µg/mL) on activity of PLC in roots of wheat seedlings under cold stress (4°C).
(DOC)

Table S5 Inhibition of cerebroside C (20 µg/mL) on activity of PLD in roots of wheat seedlings under cold stress (4°C).
(DOC)

Table S6 Effects of cerebroside C (20 µg/mL) on activity of C16:0 in roots of wheat seedlings under cold stress (4°C).
(DOC)

Table S7 Effects of cerebroside C (20 µg/mL) on contents of C18:2 in roots of wheat seedlings under cold stress (4°C).
(DOC)

Table S8 Effects of cerebroside C (20 µg/mL) on contents of C18:0 in roots of wheat seedlings under cold stress (4°C).
(DOC)

Table S9 Effects of cerebroside C (20 µg/mL) on contents of C18:3 in roots of wheat seedlings under cold stress (4°C).
(DOC)

Table S10 Effects of cerebroside C (20 µg/mL) on activity of SOD in roots of wheat seedlings under cold stress (4°C).
(DOC)

Table S11 Effects of cerebroside C (20 µg/mL) on activity of CAT in roots of wheat seedlings under cold stress (4°C).
(DOC)

Table S12 Effects of cerebroside C (20 µg/mL) on activity of GSH-Px in roots of wheat seedlings under cold stress (4°C).
(DOC)

Table S13 Effects of cerebroside C (20 µg/mL) on activity of POD in roots of wheat seedlings under cold stress (4°C).
(DOC)
