# Supplementary figures and images for: Cerebroside C Increases Tolerance to Chilling Injury and Alters Lipid Composition in Wheat Roots
Source: PLoS One. 2013 Sep 13;8(9):e73380. doi: 10.1371/journal.pone.0073380 (PMC3772805; doi:10.1371/journal.pone.0073380)

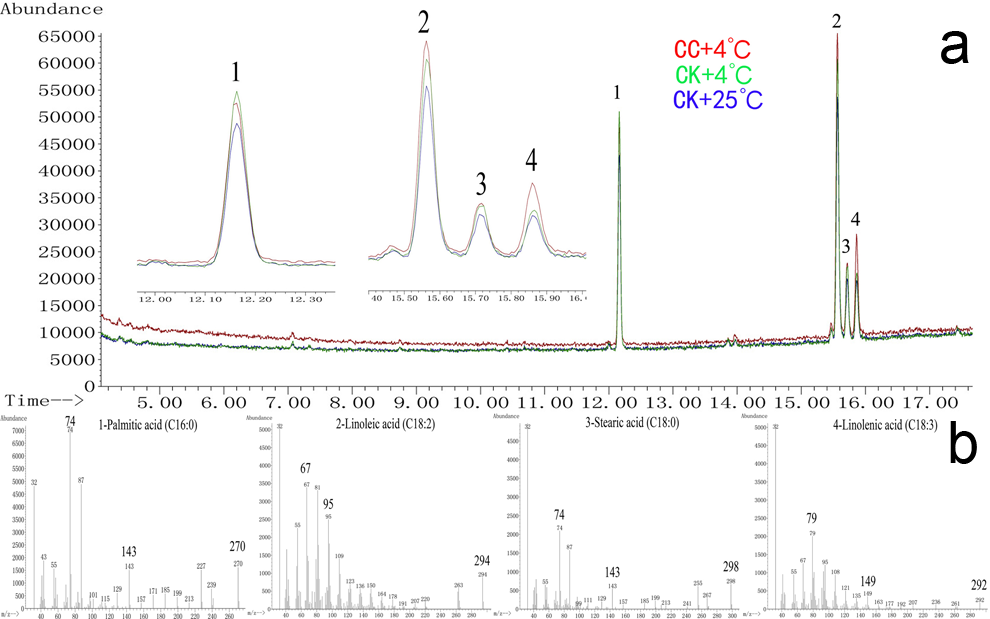

Supplement: Figure S1 — Detection of fatty acids (C16:0, C18:2, C18:0, C18:3) in roots of wheat seedlings at 48 h under cold stress by GC-MS. a. GC-MS total ion chromatogram (TIC); b. mass spectrum of the methyl esters of palmitic acid C16:0 (1), linoleic acid C18:2 (2), stearic acid C18:0 (3) and linolenic acid C18:3 (4) in roots of wheat seedlings at 48 h of cold stress. CC+4°C, pretreated with 20 µg/mL CC under cold stress; CK+4°C, treated with 0.5% DMSO under cold stress; CK+25°C, treated with 0.5% DMSO in growth chamber at 25°C. Results are expressed as the mean of three replicates (n = 3) derived from 5–10 seedlings. (TIF) [file pone.0073380.s001.tif]
