# Supplementary material for: Cerebroside C Increases Tolerance to Chilling Injury and Alters Lipid Composition in Wheat Roots
Source: PLoS One. 2013 Sep 13;8(9):e73380. doi: 10.1371/journal.pone.0073380 (PMC3772805; doi:10.1371/journal.pone.0073380)
Supplement: Table S1 — Effects of cerebroside C (20 µg/mL) on MDA content in roots of wheat seedlings under cold stress (4°C). (DOC) [file pone.0073380.s002.doc]

**Table S1** Effects of cerebroside C (20 μg/mL) on MDA content in roots of wheat seedlings under cold stress (4ºC).

| Treatments | 0 h | 6 h | 12 h | 24 h | 48 h | 72 h | 96 h |
| --- | --- | --- | --- | --- | --- | --- | --- |
| CC+4^o^C | 23.06±2.05^a^ | 25.51±1.49^a^ | 27.33±2.30^a^ | 20.79±1.75^ab^ | 20.69±0.28^b^ | 21.89±1.22^b^ | 19.87±0.90^b^ |
| CK+4^o^C | 23.76±1.22^a^ | 25.10±2.43^a^ | 41.99±0.13^b^ | 22.59±0.11^b^ | 24.02±1.70^c^ | 29.59±1.50^c^ | 28.35±1.16^c^ |
| CK+25^o^C | 23.76±1.22^a^ | 26.35±0.78^a^ | 28.90±1.35^a^ | 18.89±1.54^a^ | 16.92±1.33^a^ | 16.72±0.81^a^ | 13.10±1.14^a^ |

In each column of all tables above, the different letter indicates significant (p ≤ 0.05) difference among CC-treatment (CC+4°C), cold control (CK+4°C) and room temperature control (CK+25°C) as evaluated by Duncan’s Multiple Range Test (DMRT). Results are expressed as the mean (±) standard deviation (SD) of three replicates (n = 3) derived from 5-10 seedlings.
