# Supplementary material for: Cerebroside C Increases Tolerance to Chilling Injury and Alters Lipid Composition in Wheat Roots
Source: PLoS One. 2013 Sep 13;8(9):e73380. doi: 10.1371/journal.pone.0073380 (PMC3772805; doi:10.1371/journal.pone.0073380)
Supplement: Table S2 — Effects of cerebroside C (20 µg/mL) on RMP value in roots of wheat seedlings under cold stress (4°C). (DOC) [file pone.0073380.s003.doc]

**Table S2** Effects of cerebroside C (20 μg/mL) on RMP value in roots of wheat seedlings under cold stress (4ºC).

| Treatments | 0 h | 6 h | 12 h | 24 h | 48 h | 72 h | 96 h |
| --- | --- | --- | --- | --- | --- | --- | --- |
| CC+4^o^C | 45.58±0.68^a^ | 61.34±1.29^b^ | 61.90±2.62^a^ | 66.33±1.31^b^ | 69.02±3.01^b^ | 70.32±2.28^b^ | 67.90±2.95^b^ |
| CK+4^o^C | 49.32±1.26^b^ | 62.26±4.47^b^ | 63.31±1.05^a^ | 73.48±3.37^c^ | 79.90±2.78^c^ | 79.12±4.36^c^ | 76.68±4.69^c^ |
| CK+25^o^C | 49.32±1.26^b^ | 56.85±3.14^a^ | 58.94±3.41^a^ | 61.50±1.60^a^ | 59.65±2.90^a^ | 63.43±2.02^a^ | 63.63±2.89^a^ |

In each column of all tables above, the different letter indicates significant (p ≤ 0.05) difference among CC-treatment (CC+4°C), cold control (CK+4°C) and room temperature control (CK+25°C) as evaluated by Duncan’s Multiple Range Test (DMRT). Results are expressed as the mean (±) standard deviation (SD) of three replicates (n = 3) derived from 5-10 seedlings.
