# Supplementary material for: Cerebroside C Increases Tolerance to Chilling Injury and Alters Lipid Composition in Wheat Roots
Source: PLoS One. 2013 Sep 13;8(9):e73380. doi: 10.1371/journal.pone.0073380 (PMC3772805; doi:10.1371/journal.pone.0073380)
Supplement: Table S3 — Inhibition of cerebroside C (20 µg/mL) on activity of LOX in roots of wheat seedlings under cold stress (4°C). (DOC) [file pone.0073380.s004.doc]

**Table S3** Inhibition of cerebroside C (20 μg/mL) on activity of LOX in roots of wheat seedlings under cold stress (4ºC).

| Treatments | 0 h | 6 h | 12 h | 24 h | 48 h | 72 h | 96 h |
| --- | --- | --- | --- | --- | --- | --- | --- |
| CC+4^o^C | 5.58±0.46^a^ | 1.17±0.09^a^ | 2.67±0.47^a^ | 3.02±0.41^a^ | 2.9±0.51^a^ | 2.04±0.34^a^ | 2.39±0.15^a^ |
| CK+4^o^C | 6.46±0.35^a^ | 3.10±0.07^b^ | 3.51±0.70^a^ | 4.51±0.78^b^ | 5.25±0.35^b^ | 3.61±0.80^b^ | 3.39±0.48^b^ |
| CK+25^o^C | 6.46±0.35^a^ | 4.49±0.47^c^ | 3.11±0.80^a^ | 2.09±0.117^c^ | 2.50±0.32^a^ | 1.69±0.89^a^ | 2.04±0.37^a^ |

In each column of all tables above, the different letter indicates significant (p ≤ 0.05) difference among CC-treatment (CC+4°C), cold control (CK+4°C) and room temperature control (CK+25°C) as evaluated by Duncan’s Multiple Range Test (DMRT). Results are expressed as the mean (±) standard deviation (SD) of three replicates (n = 3) derived from 5-10 seedlings.
