# Supplementary material for: Cerebroside C Increases Tolerance to Chilling Injury and Alters Lipid Composition in Wheat Roots
Source: PLoS One. 2013 Sep 13;8(9):e73380. doi: 10.1371/journal.pone.0073380 (PMC3772805; doi:10.1371/journal.pone.0073380)
Supplement: Table S4 — Inhibition of cerebroside C (20 µg/mL) on activity of PLC in roots of wheat seedlings under cold stress (4°C). (DOC) [file pone.0073380.s005.doc]

**Table S4** Inhibition of cerebroside C (20 μg/mL) on activity of PLC in roots of wheat seedlings under cold stress (4ºC).

| Treatments | 0 h | 6 h | 12 h | 24 h | 48 h | 72 h | 96 h |
| --- | --- | --- | --- | --- | --- | --- | --- |
| CC+4^o^C | 11.36±1.86^a^ | 6.73±1.89^a^ | 6.85±1.94^a^ | 3.25±1.67^a^ | 13.86±1.44^a^ | 9.24±1.05^a^ | 10.22±1.46^a^ |
| CK+4^o^C | 16.74±0.09^b^ | 10.23±2.25^a^ | 8.35±1.69^a^ | 9.02±0.22^b^ | 18.55±1.97^b^ | 14.64±1.47^b^ | 11.82±0.83^a^ |
| CC+25^o^C | 16.74±0.09^b^ | 13.25±2.84^a^ | 8.98±3.04^a^ | 9.14±2.77^b^ | 20.59±2.21^b^ | 14.10±0.11^b^ | 12.40±1.17^a^ |

In each column of all tables above, the different letter indicates significant (p ≤ 0.05) difference among CC-treatment (CC+4°C), cold control (CK+4°C) and room temperature control (CK+25°C) as evaluated by Duncan’s Multiple Range Test (DMRT). Results are expressed as the mean (±) standard deviation (SD) of three replicates (n = 3) derived from 5-10 seedlings.
