# Supplementary material for: Cerebroside C Increases Tolerance to Chilling Injury and Alters Lipid Composition in Wheat Roots
Source: PLoS One. 2013 Sep 13;8(9):e73380. doi: 10.1371/journal.pone.0073380 (PMC3772805; doi:10.1371/journal.pone.0073380)
Supplement: Table S5 — Inhibition of cerebroside C (20 µg/mL) on activity of PLD in roots of wheat seedlings under cold stress (4°C). (DOC) [file pone.0073380.s006.doc]

**Table S5** Inhibition of cerebroside C (20 μg/mL) on activity of PLD in roots of wheat seedlings under cold stress (4ºC).

| Treatments | 0 h | 6 h | 12 h | 24 h | 48 h | 72 h | 96 h |
| --- | --- | --- | --- | --- | --- | --- | --- |
| CC+4^o^C | 8.66 ± 2.60^a^ | 3.90 ± 1.94^a^ | 4.77 ± 0.10^a^ | 10.03 ± 2.15^a^ | 8.73 ± 2.51^a^ | 10.68 ± 3.27^a^ | 15.60 ± 3.48^a^ |
| CK+4^o^C | 22.30 ± 1.36^b^ | 3.10 ± 1.62^a^ | 5.54 ± 0.38^a^ | 18.96 ± 1.38^b^ | 15.76 ± 3.96^b^ | 20.50 ± 2.31^b^ | 19.97 ± 2.97^b^ |
| CC+25^o^C | 22.30 ± 1.36^b^ | 9.86 ± 1.33^b^ | 4.72 ± 1.17^a^ | 11.34 ± 2.38^a^ | 8.12 ± 3.13^a^ | 10.52 ± 2.14^a^ | 14.02 ± 0.35^a^ |

In each column of all tables above, the different letter indicates significant (p ≤ 0.05) difference among CC-treatment (CC+4°C), cold control (CK+4°C) and room temperature control (CK+25°C) as evaluated by Duncan’s Multiple Range Test (DMRT). Results are expressed as the mean (±) standard deviation (SD) of three replicates (n = 3) derived from 5-10 seedlings.
