# Supplementary material for: Cerebroside C Increases Tolerance to Chilling Injury and Alters Lipid Composition in Wheat Roots
Source: PLoS One. 2013 Sep 13;8(9):e73380. doi: 10.1371/journal.pone.0073380 (PMC3772805; doi:10.1371/journal.pone.0073380)
Supplement: Table S6 — Effects of cerebroside C (20 µg/mL) on activity of C16:0 in roots of wheat seedlings under cold stress (4°C). (DOC) [file pone.0073380.s007.doc]

**Table S6** Effects of cerebroside C (20 μg/mL) on activity of C16:0 in roots of wheat seedlings under cold stress (4ºC).

| Treatments | 0 h | 6 h | 12 h | 24 h | 48 h | 72 h | 96 h |
| --- | --- | --- | --- | --- | --- | --- | --- |
| CC+4^o^C | 49.93 ± 0.41^a^ | 69.64 ± 0.41^a^ | 74.10 ± 0.41^a^ | 99.66 ± 0.41^a^ | 127.75 ± 0.41^a^ | 84.42 ± 0.41^a^ | 90.63 ± 0.41^a^ |
| CK+4^o^C | 60.63 ± 6.54^a^ | 79.51 ± 2.99^a^ | 111.53 ± 2.76^b^ | 137.44 ± 6.58^b^ | 161.81 ± 11.00^b^ | 99.89 ± 3.84^b^ | 150.17 ± 5.39^b^ |
| CC+25^o^C | 60.63 ± 6.54^a^ | 80.49 ± 2.02^a^ | 116.75 ± 6.28^b^ | 76.38 ± 5.98^a^ | 148.10 ± 3.14^a^ | 102.90 ± 0.41^b^ | 113.75 ± 4.45^a^ |

In each column of all tables above, the different letter indicates significant (p ≤ 0.05) difference among CC-treatment (CC+4°C), cold control (CK+4°C) and room temperature control (CK+25°C) as evaluated by Duncan’s Multiple Range Test (DMRT). Results are expressed as the mean (±) standard deviation (SD) of three replicates (n = 3) derived from 5-10 seedlings.
