# Supplementary material for: Cerebroside C Increases Tolerance to Chilling Injury and Alters Lipid Composition in Wheat Roots
Source: PLoS One. 2013 Sep 13;8(9):e73380. doi: 10.1371/journal.pone.0073380 (PMC3772805; doi:10.1371/journal.pone.0073380)
Supplement: Table S7 — Effects of cerebroside C (20 µg/mL) on contents of C18:2 in roots of wheat seedlings under cold stress (4°C). (DOC) [file pone.0073380.s008.doc]

**Table S7** Effects of cerebroside C (20 μg/mL) on contents of C18:2 in roots of wheat seedlings under cold stress (4ºC).

| Treatments | 0 h | 6 h | 12 h | 24 h | 48 h | 72 h | 96 h |
| --- | --- | --- | --- | --- | --- | --- | --- |
| CC+4^o^C | 127.99±14.80^a^ | 171.70±15.21^b^ | 221.56±9.22^c^ | 197.53±9.64^b^ | 305.23±5.05^c^ | 167.37±3.32^a^ | 192.59±4.11^a^ |
| CK+4^o^C | 110.87±5.96^a^ | 137.07±13.27^a^ | 154.24±4.90^b^ | 190.35±2.38^b^ | 254.75±8.81^b^ | 169.35±5.27^a^ | 161.36±29.64^a^ |
| CC+25^o^C | 110.87±5.96^a^ | 127.19±2.89^a^ | 199.27±5.56^b^ | 137.03±0.86^a^ | 215.82±7.61^a^ | 168.74±10.08^a^ | 149.24±1.49^a^ |

In each column of all tables above, the different letter indicates significant (p ≤ 0.05) difference among CC-treatment (CC+4°C), cold control (CK+4°C) and room temperature control (CK+25°C) as evaluated by Duncan’s Multiple Range Test (DMRT). Results are expressed as the mean (±) standard deviation (SD) of three replicates (n = 3) derived from 5-10 seedlings.
