# Supplementary material for: Cerebroside C Increases Tolerance to Chilling Injury and Alters Lipid Composition in Wheat Roots
Source: PLoS One. 2013 Sep 13;8(9):e73380. doi: 10.1371/journal.pone.0073380 (PMC3772805; doi:10.1371/journal.pone.0073380)
Supplement: Table S8 — Effects of cerebroside C (20 µg/mL) on contents of C18:0 in roots of wheat seedlings under cold stress (4°C). (DOC) [file pone.0073380.s009.doc]

**Table S8** Effects of cerebroside C (20 μg/mL) on contents of C18:0 in roots of wheat seedlings under cold stress (4ºC).

| Treatments | 0 h | 6 h | 12 h | 24 h | 48 h | 72 h | 96 h |
| --- | --- | --- | --- | --- | --- | --- | --- |
| CC+4^o^C | 159.37±10.60^a^ | 241.27±5.31^a^ | 278.98±13.16^a^ | 248.88±14.47^a^ | 284.50±4.27^a^ | 210.06±14.80^a^ | 172.69±4.03^a^ |
| CK+4^o^C | 195.98±10.74^b^ | 275.92±19.05^b^ | 304.48±4.43^b^ | 313.93±5.77^b^ | 305.74±10.66^b^ | 238.34±4.12^b^ | 193.57±5.35^b^ |
| CC+25^o^C | 195.98±10.74^b^ | 290.66±19.41^b^ | 262.29±0.44^a^ | 298.18±11.15^b^ | 288.63±16.21^a^ | 251.48±0.43^c^ | 225.74±9.80^c^ |

In each column of all tables above, the different letter indicates significant (p ≤ 0.05) difference among CC-treatment (CC+4°C), cold control (CK+4°C) and room temperature control (CK+25°C) as evaluated by Duncan’s Multiple Range Test (DMRT). Results are expressed as the mean (±) standard deviation (SD) of three replicates (n = 3) derived from 5-10 seedlings.
