# Supplementary material for: Cerebroside C Increases Tolerance to Chilling Injury and Alters Lipid Composition in Wheat Roots
Source: PLoS One. 2013 Sep 13;8(9):e73380. doi: 10.1371/journal.pone.0073380 (PMC3772805; doi:10.1371/journal.pone.0073380)
Supplement: Table S9 — Effects of cerebroside C (20 µg/mL) on contents of C18:3 in roots of wheat seedlings under cold stress (4°C). (DOC) [file pone.0073380.s010.doc]

**Table S9** Effects of cerebroside C (20 μg/mL) on contents of C18:3 in roots of wheat seedlings under cold stress (4ºC).

| Treatments | 0 h | 6 h | 12 h | 24 h | 48 h | 72 h | 96 h |
| --- | --- | --- | --- | --- | --- | --- | --- |
| CC+4^o^C | 51.89±3.26^b^ | 65.72±7.90^a^ | 81.20±7.65^b^ | 71.84±2.74^b^ | 109.30±2.24^b^ | 61.47±1.56^a^ | 60.49±6.14^a^ |
| CK+4^o^C | 37.19±1.82^a^ | 53.72±3.59^a^ | 56.77±6.60^a^ | 65.68±4.11^ab^ | 74.72±0.87^a^ | 67.40±4.51^a^ | 70.28±3.41^a^ |
| CC+25^o^C | 37.19±1.82^a^ | 65.21±5.01^a^ | 60.30±3.65^a^ | 56.40±5.88^b^ | 65.44±3.89^a^ | 68.04±14.80^a^ | 58.36±6.64^a^ |

In each column of all tables above, the different letter indicates significant (p ≤ 0.05) difference among CC-treatment (CC+4°C), cold control (CK+4°C) and room temperature control (CK+25°C) as evaluated by Duncan’s Multiple Range Test (DMRT). Results are expressed as the mean (±) standard deviation (SD) of three replicates (n = 3) derived from 5-10 seedlings.
