# Supplementary material for: Cerebroside C Increases Tolerance to Chilling Injury and Alters Lipid Composition in Wheat Roots
Source: PLoS One. 2013 Sep 13;8(9):e73380. doi: 10.1371/journal.pone.0073380 (PMC3772805; doi:10.1371/journal.pone.0073380)
Supplement: Table S10 — Effects of cerebroside C (20 µg/mL) on activity of SOD in roots of wheat seedlings under cold stress (4°C). (DOC) [file pone.0073380.s011.doc]

**Table S10** Effects of cerebroside C (20 μg/mL) on activity of SOD in roots of wheat seedlings under cold stress (4ºC).

| Treatments | 0 h | 6 h | 12 h | 24 h | 48 h | 72 h | 96 h |
| --- | --- | --- | --- | --- | --- | --- | --- |
| CC+4^o^C | 5753.87±537.41^b^ | 8373.49±508.74^b^ | 8098.46±245.42^b^ | 8279.23±652.83^b^ | 7099.83±308.76^b^ | 7120.42±108.21^b^ | 6784.36±52.04^a^ |
| CK+4^o^C | 4559.85±78.45^a^ | 7054.46±115.19^a^ | 7519.70±128.98^a^ | 5668.38±353.15^a^ | 6182.10±159.61^a^ | 6526.79±62.34^a^ | 5903.27±62.34^a^ |
| CC+25^o^C | 4559.85±78.45^a^ | 6838.97±527.97^a^ | 7236.15±459.61^a^ | 5955.85±338.14^a^ | 6898.75 ± 2.94^b^ | 6721.30±253.64^ab^ | 6413.30±358.82^a^ |

In each column of all tables above, the different letter indicates significant (p ≤ 0.05) difference among CC-treatment (CC+4°C), cold control (CK+4°C) and room temperature control (CK+25°C) as evaluated by Duncan’s Multiple Range Test (DMRT). Results are expressed as the mean (±) standard deviation (SD) of three replicates (n = 3) derived from 5-10 seedlings.
