# Supplementary material for: Cerebroside C Increases Tolerance to Chilling Injury and Alters Lipid Composition in Wheat Roots
Source: PLoS One. 2013 Sep 13;8(9):e73380. doi: 10.1371/journal.pone.0073380 (PMC3772805; doi:10.1371/journal.pone.0073380)
Supplement: Table S11 — Effects of cerebroside C (20 µg/mL) on activity of CAT in roots of wheat seedlings under cold stress (4°C). (DOC) [file pone.0073380.s012.doc]

**Table 11** Effects of cerebroside C (20 μg/mL) on activity of CAT in roots of wheat seedlings under cold stress (4ºC).

| Treatments | 0 h | 6 h | 12 h | 24 h | 48 h | 72 h | 96 h |
| --- | --- | --- | --- | --- | --- | --- | --- |
| CC+4^o^C | 46.72±3.38^b^ | 37.51±2.61^a^ | 146.18±2.46^b^ | 48.34±5.96^a^ | 78.14±1.56^a^ | 49.72±4.23^b^ | 50.90±6.89^a^ |
| CK+4^o^C | 26.90±3.73^a^ | 34.78±1.14^a^ | 115.50±15.21^a^ | 37.38±3.04^a^ | 65.51±7.77^a^ | 36.04±1.37^a^ | 49.24±4.63^a^ |
| CC+25^o^C | 26.90±3.73^a^ | 27.29±7.50^a^ | 100.49±5.03^a^ | 32.24±1.10^a^ | 72.30±1.19^ab^ | 49.37±10.35^b^ | 68.37±7.33^a^ |

In each column of all tables above, the different letter indicates significant (p ≤ 0.05) difference among CC-treatment (CC+4°C), cold control (CK+4°C) and room temperature control (CK+25°C) as evaluated by Duncan’s Multiple Range Test (DMRT). Results are expressed as the mean (±) standard deviation (SD) of three replicates (n = 3) derived from 5-10 seedlings.
