# Supplementary material for: Cerebroside C Increases Tolerance to Chilling Injury and Alters Lipid Composition in Wheat Roots
Source: PLoS One. 2013 Sep 13;8(9):e73380. doi: 10.1371/journal.pone.0073380 (PMC3772805; doi:10.1371/journal.pone.0073380)
Supplement: Table S12 — Effects of cerebroside C (20 µg/mL) on activity of GSH-Px in roots of wheat seedlings under cold stress (4°C). (DOC) [file pone.0073380.s013.doc]

**Table S12** Effects of cerebroside C (20 μg/mL) on activity of GSH-Px in roots of wheat seedlings under cold stress (4ºC).

| Treatments | 0 h | 6 h | 12 h | 24 h | 48 h | 72 h | 96 h |
| --- | --- | --- | --- | --- | --- | --- | --- |
| CC+4^o^C | 2660.31±65.71^a^ | 1169.82±26.87^a^ | 6236.49±220.04^b^ | 3527.03±158.52^a^ | 8098.84±265.81^b^ | 1939.88±181.74^b^ | 2635.34±299.31^a^ |
| CK+4^o^C | 2262.99±94.78^a^ | 1257.99±70.95^a^ | 3013.43±335.80^a^ | 3061.92±430.47^a^ | 5466.83±429.73^a^ | 697.62±3398.1^a^ | 3136.17±16.22^b^ |
| CC+25^o^C | 2262.99±94.78^a^ | 1515.29±108.03^a^ | 2509.85±55.16^a^ | 2690.16±268.83^a^ | 6241.55±325.20^a^ | 659.68±70.97^a^ | 2727.80±363.38^a^ |

In each column of all tables above, the different letter indicates significant (p ≤ 0.05) difference among CC-treatment (CC+4°C), cold control (CK+4°C) and room temperature control (CK+25°C) as evaluated by Duncan’s Multiple Range Test (DMRT). Results are expressed as the mean (±) standard deviation (SD) of three replicates (n = 3) derived from 5-10 seedlings.
