# Supplementary material for: Cerebroside C Increases Tolerance to Chilling Injury and Alters Lipid Composition in Wheat Roots
Source: PLoS One. 2013 Sep 13;8(9):e73380. doi: 10.1371/journal.pone.0073380 (PMC3772805; doi:10.1371/journal.pone.0073380)
Supplement: Table S13 — Effects of cerebroside C (20 µg/mL) on activity of POD in roots of wheat seedlings under cold stress (4°C). (DOC) [file pone.0073380.s014.doc]

**Table S13** Effects of cerebroside C (20 μg/mL) on activity of POD in roots of wheat seedlings under cold stress (4ºC).

| Treatments | 0 h | 6 h | 12 h | 24 h | 48 h | 72 h | 96 h |
| --- | --- | --- | --- | --- | --- | --- | --- |
| CC+4^o^C | 868.31±47.17^a^ | 833.25±22.35^a^ | 789.44±29.58^a^ | 860.55±47.98^a^ | 1087.97±8.91^a^ | 977.97±29.42^a^ | 980.19±12.64^a^ |
| CK+4^o^C | 876.25±4.78^a^ | 833.25±14.42^a^ | 774.17±51.50^a^ | 802.06±8.83^a^ | 1132.30±23.72^a^ | 950.78±15.17^a^ | 958.12±43.90^a^ |
| CC+25^o^C | 876.25±4.78^a^ | 824.22±24.31^a^ | 774.72±10.68^a^ | 898.06±17.13^a^ | 1016.16±41.46^ab^ | 914.98±30.65^a^ | 847.80±11.40^b^ |

In each column of all tables above, the different letter indicates significant (p ≤ 0.05) difference among CC-treatment (CC+4°C), cold control (CK+4°C) and room temperature control (CK+25°C) as evaluated by Duncan’s Multiple Range Test (DMRT). Results are expressed as the mean (±) standard deviation (SD) of three replicates (n = 3) derived from 5-10 seedlings.
